# Supplementary material for: A randomized clinical trial of vitamin D3 (cholecalciferol) in ulcerative colitis patients with hypovitaminosis D3
Source: PeerJ. 2017 Aug 3;5:e3654. doi: 10.7717/peerj.3654 (PMC5545112; doi:10.7717/peerj.3654)
Supplement: Supplemental Information 1 [file peerj-05-3654-s001.docx]

**Vitamin D_3_ 2,000 IU/day for ninety days**

| **UC Patient** | **Vitamin D levels** | | | **Patial Mayo Score (0-9)** | | | | | | **SIBDQ score (1-7)** | | | | |
| --- | --- | --- | --- | --- | --- | --- | --- | --- | --- | --- | --- | --- | --- | --- |
|  | Day 0 | Day 90 | Change | Day 0 | | Day 90 | Change | | Day 0 | | | Day 90 | | Change |
| 1 | 19 | 18 | - 1 | 3 | 1 | | | -2 | | 5 | 5 | | 0.0 | |
| 2 | 10 | 13 | 3 | 2 | 0 | | | -2 | | 6.3 | 5.8 | | -0.5 | |
| 3 | 15 | 22 | 7 | 1 | 1 | | | 0 | | 4.9 | 4.4 | | -0.5 | |
| 4 | 28 | 37 | 9 | 1 | 3 | | | 2 | | 4.9 | 3.4 | | -1.5 | |
| 5 | 17 | 26 | 9 | 3 | 1 | | | -2 | | 5.5 | 5.9 | | 0.4 | |
| 6 | 18 | 19 | 1 | 1 | 0 | | | -1 | | 5.7 | 7.8 | | 2.1 | |
| 7 | 14 | 18 | 4 | 0 | 0 | | | 0 | | 6.4 | 6.7 | | 0.3 | |
| 8 | 15 | 23 | 8 | 0 | 1 | | | 1 | | 5.7 | 5.9 | | 0.2 | |

**Vitamin D_3_ 4,000 IU/day for ninety days**

| **UC Patient** | **Vitamin D levels** | | | **Partial Mayo scores (0-9)** | | | **SIBDQ Score(1-7)** | | |
| --- | --- | --- | --- | --- | --- | --- | --- | --- | --- |
|  | Day 0 | Day 90 | Change | Day 0 | Day 90 | Change | Day 0 | Day 90 | Change |
| 1 | 18 | 36 | 18 | 6 | 5 | - 1 | 4.5 | 4.8 | 0.3 |
| 2 | 12 | 18 | 6 | 3 | 5 | 2 | 5.4 | 6.1 | 0.7 |
| 3 | 11 | 29 | 18 | 6 | 1 | - 5 | 5.2 | 6.4 | 1.2 |
| 4 | 6 | 21 | 15 | 2 | 3 | 1 | 4.9 | 5.1 | 0.2 |
| 5 | 18 | 23 | 5 | 5 | 7 | 2 | 6.2 | 6.4 | 0.2 |
| 6 | 20 | 33 | 13 | 1 | 0 | - 1 | 6.5 | 6.2 | - 0.3 |
| 7 | 11 | 43 | 32 | 8 | 3 | - 5 | 1.7 | 4.2 | 2.5 |
| 8 | 15 | 31 | 16 | 2 | 3 | 1 | 5.6 | 5.8 | 0.2 |
| 9 | 16 | 29 | 13 | 2 | 0 | - 2 | 3.8 | 6.0 | 2.2 |
| 10 | 16 | 48 | 32 | 5 | 0 | - 5 | 4.0 | 6.2 | 2.2 |
