# Supplementary material for: A randomized clinical trial of vitamin D3 (cholecalciferol) in ulcerative colitis patients with hypovitaminosis D3
Source: PeerJ. 2017 Aug 3;5:e3654. doi: 10.7717/peerj.3654 (PMC5545112; doi:10.7717/peerj.3654)
Supplement: Supplemental Information 2 [file peerj-05-3654-s002.docx]

**TITLE:** Supplementation of vitamin D_3_ (Cholecalciferol) in patients with inflammatory bowel diseases (IBD) and hypovitaminosis D: A prospective randomized trial.

**INVESTIGATORS:** Jagrati Mathur, Paul Mills, Soe Naing, David Limsui

**BACKGROUND:**

Crohns disease (CD) and Ulcerative colitis (UC) are the main forms of inflammatory bowel disease (IBD). The pathogenesis of IBD is incompletely understood. It is thought to involve a complex interplay of genetic, environmental and microbial factors in the context of an abnormal and inappropriate autoimmune response, when defective mucosa is exposed to unknown luminal antigens. Autoimmune T cells attack the bowel mucosa and cause inflammation by releasing inflammatory cytokines such as interleukins (IL-17), Interferon- gamma (INF) and a few other regulatory cells. Another potential pathogenic factor in CD is impaired mucosal barrier function and intestinal hyperpermeability. _(1)_

The incidence of hypovitaminosis D has been reported to be as high as 75%, in patients with IBD. _(2)_ However, it is unclear whether low vitamin D levels contribute to the pathogenesis of IBD or are a consequence of IBD. Recent animal data however does suggest that maintenance of epithelial barrier integrity in the large intestine by vitamin D is critical in preventing IBD. _(3)_

The sum of vitamin D_2_ and D_3_ contributes to the total body stores of vitamin D. Vitamin D synthesis mostly takes place endogenously in the skin by sunlight (UV) exposure and 7- dehydrocholesterol gets converted to Cholecalciferol photochemically. Cholecalciferol is then converted by the liver into 25 hydroxy cholecalciferol 25(OH)D_,_ which is the major circulatory form of vitamin D. 25(OH)D is largely inactive and has a long half life and thus is the best indicator of overall vitamin D status. Finally 25(OH)D is then converted in the kidney to its active form 1,25(OH)_2_D_3_ (D_3_ or calcitriol). Although vitamin D can also be absorbed by the intestine but most foods contain insignificant amounts of vitamin D except oily fish.

Based on the 25(OH)D_3_ levels in an individual, vitamin D may be adequate or deficient in the body. Levels <10 ng/ml is ‘severe vitamin D deficiency’, levels between 10-20ng/ml is ‘vitamin D deficiency’, levels between 20-30ng/ml is ‘vitamin D insufficiency’ and levels >30 ng/ml are considered ‘adequate vitamin D’. According to the 2011 Institute of Medicine (IOM) report, the required daily allowance (RDA) of vitamin D to achieve 25(OH)D levels >20ng/ml is between 600 IU to 4,000IU in patients btw 1-70 yrs and >71yrs of age is 800IU- 4,000IU. _(4)_

In the last 5 years, there has been an increasing interest in the role of vitamin D and its analogues as immune modulators, in addition to their role in the metabolism of calcium and phosphorous and in bone formation and mineralization. The immune regulatory role of Vitamin D affects both the innate and adoptive immunity and contributes to the immune-tolerance of self-structures.

Supplementation of Vitamin D in animals has also shown to reduce the severity of symptoms and exacerbations in autoimmune diseases such as rheumatoid arthritis and Multiple Sclerosis in other studies. _(5,6,7)_ Studies show that low levels of vitamin D are independently associated with a lower quality of life and greater disease activity in patients with Crohns _(2, 8)_ . In a prospective study involving 37 crohns patients, the investigators compared the effect of active vitamin D (1,25 (OH)D)(aVD) versus plain vitamin D (25 (OH)D)(pVD) on bone metabolism and disease activity and showed that both the forms of vitamin D significantly decreased disease activity and markers of bone turnover. _(9)_ A recent randomized double blind placebo-control study, with 108 crohns patients, showed that supplementation of oral Vitamin D_3_ (1200 IU) led to significant improvement in the serum vitamin D levels in 3 months and reduced the risk of relapses (29% to 13%) compared to the placebo group, however this did not reach clinical significance. _(10)_

However, more data is required to make conclusions regarding the effect of vitamin D_3_ supplementation in patients with IBD. Furthermore, there is no clear consensus regarding the appropriate dose of vitamin D supplementation in IBD patients. _(11)_

Therefore, in this trial we propose to evaluate the effect of Vitamin D supplementation on IBD disease activity and to evaluate the optimal dose of this vitamin in IBD patients who are deficient.

**HYPOTHESIS:** Supplementation of vitamin D in IBD patients with hypovitaminosis D can improve their quality of life and decrease the IBD disease activity scores.

**OBJECTIVES**:

**1)** To determine the appropriate dose of vitamin D3 supplementation in patients with IBD and hypovitaminosis D.

**2)** To assess the effect of vitamin D3 supplementation on the disease activity and quality of life in patients with IBD and hypovitaminosis D.

**DESIGN AND SETTING:**

This is a Prospective Randomized controlled trial which will be conducted at the Derian Koligian ambulatory care center (ACC), the University medical Specialty centers for Gastroenterology and Community regional Medical center (CRMC), Fresno, CA. The recruitment period will be for the period of a year i.e. May 2012-Aprill 2013. This is an interdepartmental study among the departments of Internal medicine, Endocrinology and Gastroenterology.

**INCLUSION:**

Patients with IBD > 18yrs of age, with 25(OH)D levels <30ng/ml, who can give written consent will be eligible for enrollment.

**EXCLUSION:**

1. Age <18yrs
2. Pregnancy
3. Patients on Vitamin D supplementation >2,000 IU/daily prior to study enrollment.

**METHODS:**

Patients with a history of inflammatory bowel disease (IBD) seen at the at the Derian Koligian ambulatory care center (ACC), the University for Gastroenterology and Hepatology Associates and at the Community Regional Medical Center (CRMC), Fresno, CA from May 2012- April 2013 will routinely get screened for vitamin D deficiency with a 25(OH) D level, as a standard of care in these patients. Those patients with IBD and low vitamin D levels i.e. 25(OH)D levels <30 ng/ml will be eligible for enrollment in this study. The diagnosis of IBD will be confirmed either by a prior endoscopy, pathology, radiographic imaging or on the basis of IBD treatment. IBD patients already on vitamin D_3_ supplementation >2,000IU per day will be excluded. Once identified, the IBD patients with low vitamin D levels will be referred to the study sub-investigators or the research coordinator for possible enrollment. A flyer will also be put up in these outpatient clinics to create awareness about the study. All eligible patients need a written consent to participate in the study.

A trained research coordinator will be informed of potential IBD patients eligible for enrollment coming to the ACC clinic, University of Gastroenterology and Hepatology Associate clinic and the CRMC hospital either by the gastroenterology clinic doctors or fellows or the study investigators and potential patients will also be identified by screening the outpatient gastrointestinal (GI) clinic schedules at the ACC for potential IBD patients. The research coordinator once informed about a potential IBD patient with hypovitaminosis D, will screen and then contact these eligible IBD patients to ask if they are willing to take part in the study. If the patient is agreeable to be a part of the study, the research coordinator will ask them to come to the ACC clinic to consent, complete a data collection form, and get blood work. The investigators will then ask the enrolled subject to fill out 2 questionnaires- one for the IBD activity score and another will be for quality of life. The investigators at the time of enrollment will also dispense prepackaged vitamin D_3_ supplements.

A total of 30 patients will be included in this study during the one-year study period. The enrolled patients will be randomized using a permuted-block randomization procedure to insure equal sample sizes into the two vitamin D_3_ dose arms of the trail, either 2,000IU or 4,000IU.

For our study, the outpatient pharmacy at ACC will preorder a 3 month supply of the vitamin D_3_ supplements from a brand called ‘Major’ for all 30 patients. The outpatient pharmacy at the ACC will prepackage, label and number each of the packages containing a 3 month supply of vitamin D_3,_ for the 30 patients enrolled. Among the 30 vitamin D_3_ packages prepared by the pharmacy- 15 packages will contain 2,000IU (1 bottle) of vitamin D _3_ and the other 15 packages will contain 4,000IU (2 bottles) of vitamin D_3_. The vitamin D_3_ packages will be randomly distributed among the 30 enrolled patients such that half of the patients receive 2,000IU/day and the other half will receive 4,000 IU/day. The vitamin D_3_ bottles in these packages will be labeled such that they will not indicate the dose of vitamin D but will have directions on how to take the vitamin D_3_ daily. Thus, the enrolled patients will be unaware of the vitamin D dose they will be receiving for the next 3 months. All these packages prepared by the pharmacy will have number and a master key made by the pharmacy, will contain a list of all the vitamin D package numbers and the corresponding vitamin D doses available in these packages. The investigators will keep the master key but will not be aware of how much of vitamin D_3_ each patient will be receiving while dispensing the vitamin D_3_ or during the period of enrollment into the study. This is to prevent bias of the investigator while filling out the IBD questionnaires. The vitamin D prepackages prepared by the pharmacy will be stored in the medical supply room at the ACC, which are locked doors that can only be open by a security code. Compliance will be evaluated by patient interview and by a pill count of the remaining tablets on the last visit.

All patients will need to get blood work done (listed below) and also fill out validated IBD questionnaires for quality of life assessment and IBD related disease activity scores, at the start of the study and at the end of 3 months. The following blood work will be drawn and analyzed at the community regional medical centers outpatient lab:

- 1. 25 hydroxy vitamin D levels
  2. Complete Blood Count (CBC) with differential
  3. Complete metabolic panel (CMP)
  4. Serum phosphorous
  5. Parathyroid hormone (PTH)
  6. Erythrocyte sedimentation rate (ESR)
  7. C- reactive protein (CRP)

A general data collection form will be completed and two validated questionnaires regarding quality of life and IBD disease activity (attached) will completed by all enrolled patients at the beginning of the study and at the end of the study period i.e. 3 months. _(12- 14)_

These forms include:

- Data collection form (Form 1)
- Quality of life - Short IBD Questionnaire score (SIBDQ) for all IBD patients (Questionnaire 1)
- Mayo score for UC patients (Questionnaire 2)
- Crohn’s Disease Activity Index (UCDAI) for CD patients (Questionnaire 3)

At the end of the study, we will compare the scores of the above questionnaire to the previous scores to assess the differences in the IBD disease activity and quality of life before and after the supplementation of vitamin D_3_. For patients with UC, the partial mayo score (validated) will be used which includes clinical criteria only (stool frequency, rectal bleeding, physician global assessment score) and not endoscopic scores and we will use a UCDAI form for clinical assessment of CD disease activity.

**Study Plan:**

| **Day** | **Plan** |
| --- | --- |
| Enrollment into the study | Subject consents to be part of the study, gets blood tests, fill out a data collection form and IBD questionnaires. The subjects then receive a package containing a 3 month supply of vitamin D_3_. |
| Day 1 of treatment | Begin taking vitamin D_3_ once a day for 3 months and keep taking it until asked to stop by the investigators. |
| Day 1-90 | Keep taking the vitamin D once a day. Patients can stop taking the vitamin D if they have bad side effects or the patient’s IBD gets worse. |
| Day 90 (3 months) | Subject returns to the doctor's office to repeat the blood tests and fill out the IBD questionnaires. Compliance will be assessed in this visit. |

| IBD patients seen at the GI outpatient clinics get screened for vitamin D deficiency |
| --- |


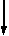


| **Patients with IBD and vitamin D deficiency are identified and then screened**  **for enrollment into the study** |
| --- |


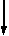


| **At the time of enrollment into the study:**  **Blood draws for labs and 2 questionnaires completed**  **and**  **All patients receive *Vitamin D supplementation*** |
| --- |


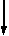


| ***Randomize***  ***(You will be in Group 1 or Group 2)*** |
| --- |


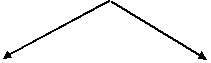


| \| *Group 1*  ***Vitamin D 2,000 IU for 3 months*** \| \| --- \| |  | \| *Group 2*  ***Vitamin D 4,000 IU for 3 months*** \| \| --- \| |
| --- | --- | --- | --- | --- |

**At the end of 3 months, you will be asked to come in for repeat:**

- **Blood work**
- **Questionnaires- IBD activity, Quality of life**
- **Pill counting to assess compliance**

**RISKS/DISCOMFORT:**

1. Blood draw- can cause infection/ bleeding/hematoma at the venipuncture site.
2. Vitamin D supplementation--- No adverse outcomes are expected as 4,000IU/day is considered physiological dose and the general public can purchase up to 50,000IU/day over the counter without monitoring.

Published cases of toxicity, for which serum levels and dose are known, all involve intake of ≥ 40,000 IU (1000 mcg) per day. (13) Two different cases involved intake of over 2,000,000 IU per day - both men survived. (14,15) Upper limit for a substance is the amount up to which is considered safe and without risk of adverse effects in the majority of the population. Toxicity threshold for a substance is the amount beyond which symptoms of toxicity manifest.

These values for vitamin 25(OH)D are as follows:

- Toxicity threshold level - 200-250 ng/mL (500-750 nmol/L) (16,17,18,19,20,21)
- Upper limit - 100 ng/mL (250 nmol/L)

The large range between 25(OH)D’s upper limit and its threshold value implies a degree of safety at serum levels up to 100 ng/mL (250 nmol/L), since concentrations twice this amount have yet to ever be associated with toxicity. (16) In animal models, serum concentrations have reached as high as 400-700 ng/mL (1,000-1750 nmol/L) before toxic effects (severe [hypercalcemia](http://www.vitamindcouncil.org/mod_cms/glossary/view_list.aspx?l=H#Hypercalcemia)) were observed. (20, 21 )

The first sign of vitamin D toxicity is hypercalcuria (excess calcium in the urine) followed by hypercalcemia (high blood calcium). The following symptoms may present:

- nausea
- vomiting
- poor appetite
- constipation (possibly alternating with diarrhea)
- weakness
- weight loss
- tingling sensations in the mouth
- confusion
- heart rhythm abnormalities

The immediate symptoms of vitamin D overdose are:

- Abdominal cramps
- Nausea
- Vomiting

**STUDY ANALYSIS:**

The PI will design and implement an Excel- based data collection form. The form will be pre-tested on ten patients prior to actual data collection. Upon data collection completion, the data will be analyzed using SPSS. Continuous variables will be examined for normality and if assumption is met, differences in mean values will be tested using Student’s t test an analysis of variance (ANOVA). If not normally distributed, non-parametric procedures will be used, including Wilcoxan rank Sum test. Categorical data will be analyzed using Fisher’s exact test and Chi square analysis. Since before/after comparisons will be performed on the same study patients we will utilize paired t tests and McNemar’s chi-square test.

**Power:** It is anticipated that we will only be able to accrue a total of 30 patients in this study, thereby limiting our statistical power. However a recent case–control study based on only 34 cases of IBD and 34 controls was able to demonstrate statistically significant differences in many of the same parameter we wish to test.

**Form 1:**

**Data Collection Form**

1)     Age: ___________ (yrs)

2)     Sex: Male / Female

3)     Race: Caucasian/ Hispanic/ Black/ Asian/ Indian/ other:

4)     Weight: _______ (lb)

5)     Height: _____ (m)

6)     BMI (weight/height^^2^): _________()

7)     History of smoking-

     Current / former/ nonsmoker

8)     List of medications:

- Current IBD medications:
- Prior IBD medications:
- Last steroid use:
- Other current medications:

9)     Type of IBD- CROHNS/ ULCERATIVE COLITIS / INDETERMINATE

10)     Duration of Inflammatory bowel Disease- IBD: _____ (yrs)

11)      Location/ subtype of IBD:

     Upper GI/ Jejuno-ileitis / Ileitis / Ileo-colitis/ Colitis/  Proctitis

12)    Have you had a small bowel resection in the past- Yes/No

     If yes; what type of surgery?____________________________

15)     Are you currently on any vitamin D supplementation: Yes/ No

- If yes; How much? ______________ IU

16)     Type of Occupation: ___________ ; mostly Indoor/ outdoor/ mixed

17)     Sun exposure- No of hours/day in the sun:

Average number of days per week with >2 midday hours exposure to sunlight during summer:


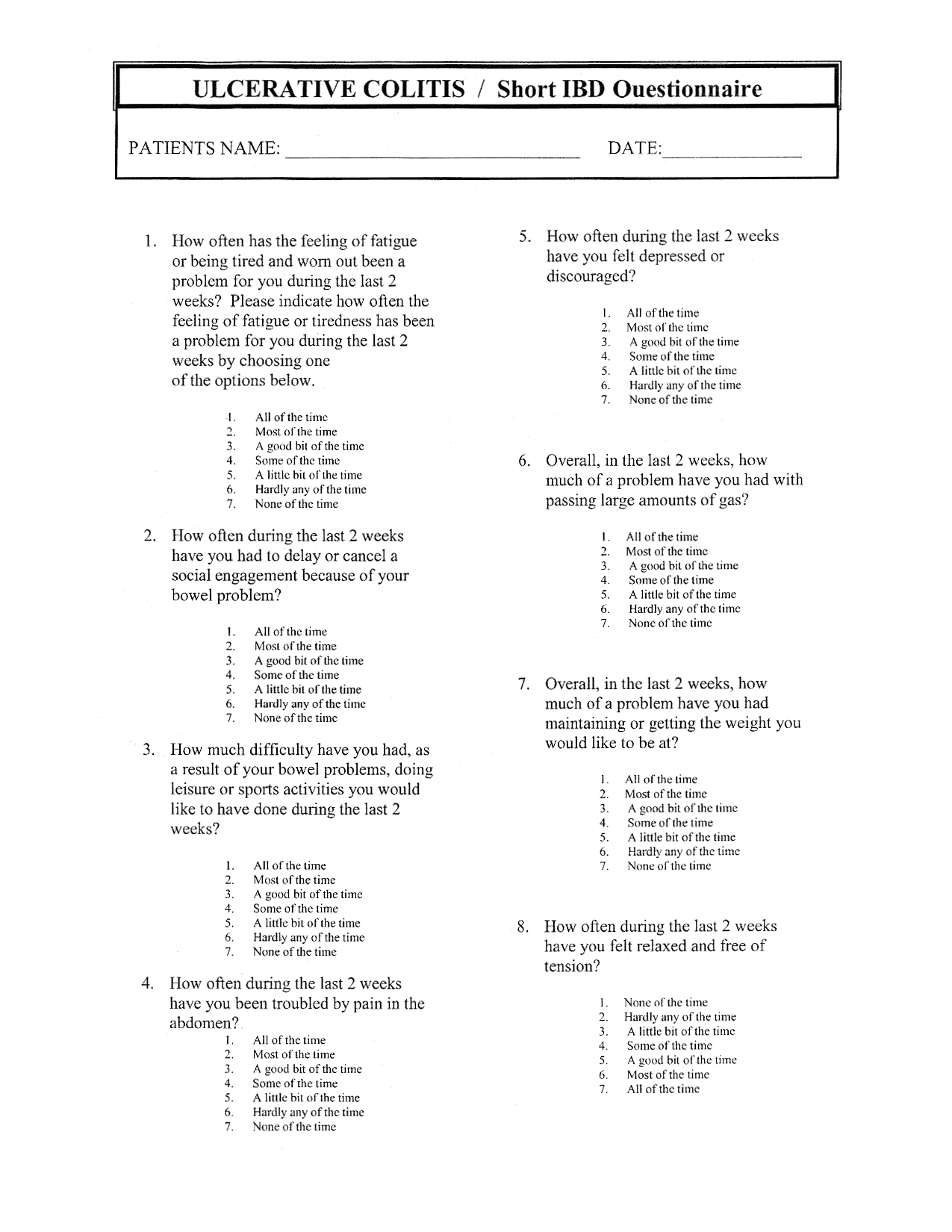
**Questionnaire 1: Short IBD Quality of life tool**

**
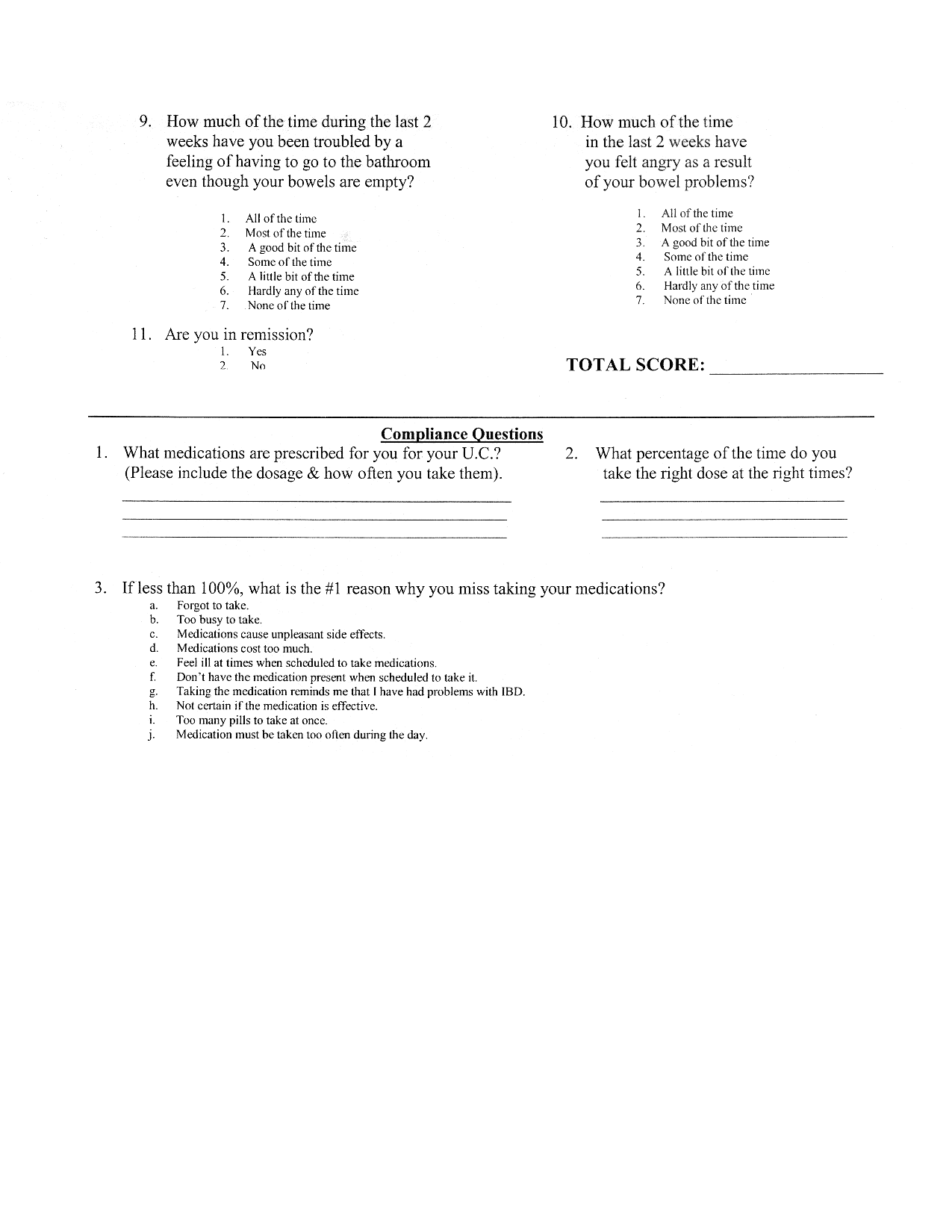
**

**Questionnaire 2:**

| **Mayo Scoring System for Assessment of Ulcerative Colitis Activity :** (22)  **Stool frequency**  0 = Normal no. of stools for this patient  1 = 1 to 2 stools more than normal  2 = 3 to 4 stools more than normal  3 = 5 or more stools more than normal  Subscore, 0 to 3=  **Rectal bleeding‡**  0 = No blood seen  1 = Streaks of blood with stool less than half the time  2 = Obvious blood with stool most of the time  3 = Blood alone passes  Subscore, 0 to 3=  **Findings on endoscopy**  0 = Normal or inactive disease  1 = Mild disease (erythema, decreased vascular pattern, mild friability)  2 = Moderate disease (marked erythema, lack of vascular pattern, friability,  erosions)  3 = Severe disease (spontaneous bleeding, ulceration)  Subscore, 0 to 3=  **Physician’s global assessment**  0 = Normal  1 = Mild disease  2 = Moderate disease  3 = Severe disease  Subscore, 0 to 3=  The Mayo score ranges from 0 to 12, with higher scores indicating more severe  disease.  **Questionnaire 3:**  **Crohns Disease activity Index (CDAI) for patients with Crohns:** (23)  Link to online calculator is: <a href="http://www.ibdjohn.com/cdai/"> Crohn's Disease Activity Index, CDAI </a>  **Clinical or laboratory variable** |  |
| --- | --- |


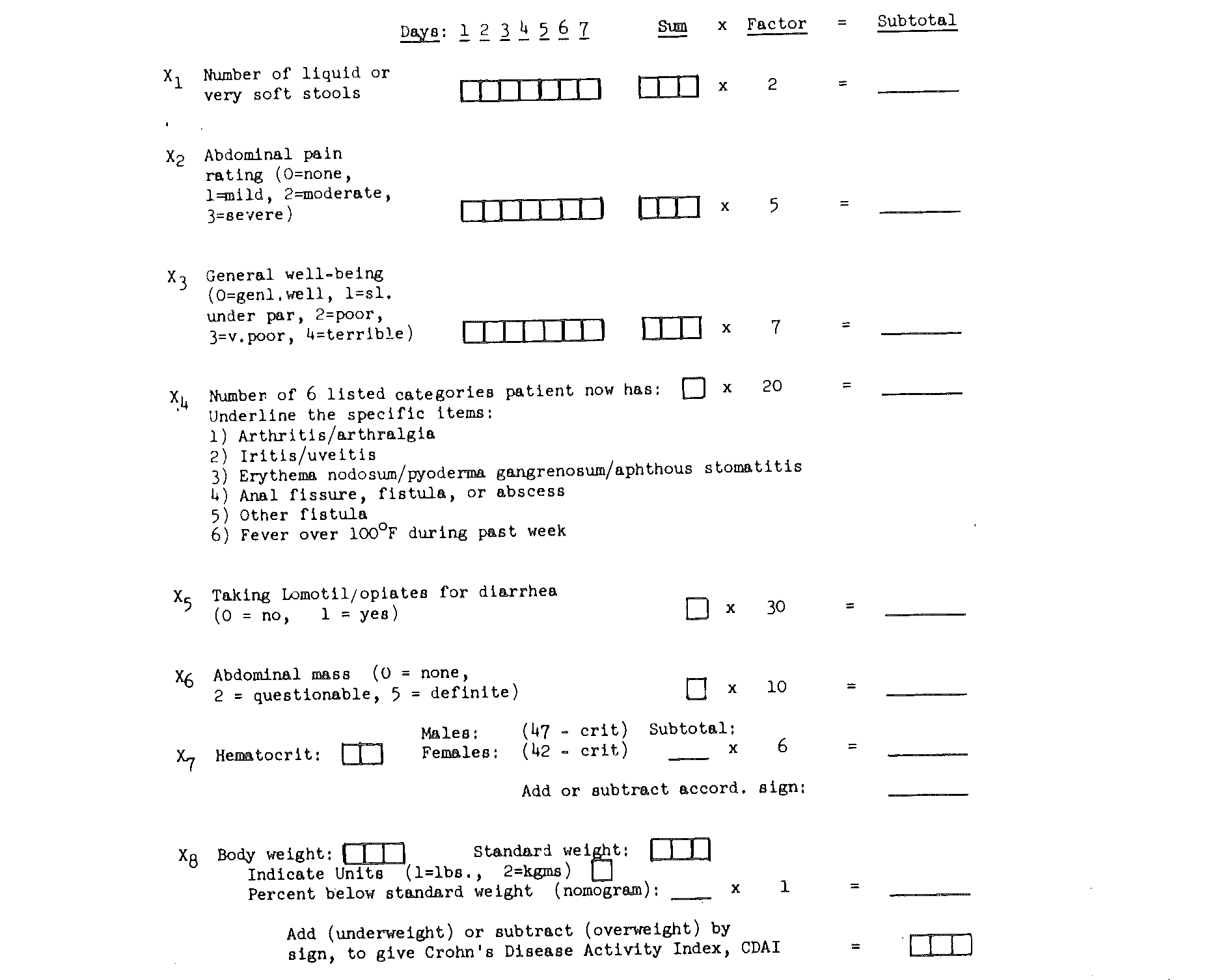


**REFERANCES**:

1. (Gibson, 2004)
2. Ulitsky, A., Ananthakrishnan A.N., Naik A., Skaros S., Zadvornova Y., Binion D.G., Issa M.,(2011)Vitamin D deficiency in patients with inflammatory bowel disease: Association with disease Activity and quality of life. Journal of parenteral and Enteral nutrition 35:308_316
3. Kong et al 2008
4. Ross AC, Manson JE, Abrams SA *et al*. The 2011 Report on dietary reference intakes for calcium and vitamin D from the Institute of Medicine: what clinicians need to know. *J Clin Endocrinol Metab* 2011; **96**: 53–58.
5. Andjelkovic, Z., Vojinovic, J., Pejnovic, N., Popovic, M., Dujic, A., Mitrovic, D. et al. (1999) Disease modifying and immune modulatory effects of high dose 1 alpha (OH) D3 in rheumatoid arthritis patients. Clin Exp Rheumatol 17: 453_456.
6. Nordvik, I., Myhr, K.M., Nyland, H. and Bjerve, K.S. (2000) Effect of dietary advice and n-3 supplementation in newly diagnosed MS patients. *Acta Neurol Scand* 102: 143_149.
7. Goldberg, P., Fleming, M.C. and Picard, E.H. (1986) Multiple sclerosis: Decreased relapse rate through dietary supplementation with calcium, magnesium and vitamin D. Med Hypotheses 21: 193_200.
8. Joseph AK, et al. 25(OH) vitamin D level in Crohn’s disease: association with sun exposure and disease activity 2009.    *Indian J Med Research* 130: 133-137
9. Miheller et al 2009
10. Jorgenson et al 2010
11. Ross AC, Manson JE, Abrams SA *et al*. The 2011 Report on dietary reference intakes for calcium and vitamin D from the Institute of Medicine: what clinicians need to know. *J Clin Endocrinol Metab* 2011; **96**: 53–58.
12. [Irvine EJ](https://vpn.ucsf.edu/,DanaInfo=www.ncbi.nlm.nih.gov+pubmed?term=%22Irvine%20EJ%22%5BAuthor%5D), [Zhou Q](https://vpn.ucsf.edu/,DanaInfo=www.ncbi.nlm.nih.gov+pubmed?term=%22Zhou%20Q%22%5BAuthor%5D), [Thompson AK](https://vpn.ucsf.edu/,DanaInfo=www.ncbi.nlm.nih.gov+pubmed?term=%22Thompson%20AK%22%5BAuthor%5D). The Short Inflammatory Bowel Disease Questionnaire: a quality of life instrument for community physicians managing inflammatory bowel disease. CCRPT Investigators. Canadian Crohn's Relapse Prevention Trial. [Am J Gastroenterol.](https://vpn.ucsf.edu/pubmed/,DanaInfo=www.ncbi.nlm.nih.gov+8759664) 1996 Aug;91(8):1571-8. Department of Medicine, McMaster University, Hamilton, Ontario.
13. Schroeder KW, Tremaine WJ, Ilstrup DM. Coated oral 5-aminosalicylic acid therapy for mildly to moderately active ulcerative colitis: a randomized study. N Engl J Med 1987;317:1625-9.
14. Best WR, Becktel JM, Singleton JW, Kern F Jr. (Mar 1976). "Development of a Crohn's disease activity index. National Cooperative Crohn's Disease Study". *Gastroenterology* **70** (3): 439–444. [PMID](http://en.wikipedia.org/wiki/PubMed_Identifier) [1248701](http://www.ncbi.nlm.nih.gov/pubmed/1248701)

[Remission](http://en.wikipedia.org/wiki/Remission) of Crohn's disease is defined as a fall in the CDAI of less than 150. Severe disease is defined as a value of greater than 450. Most major research studies on medications in Crohn's disease define response as a fall of the CDAI of greater than 70 points.

**REFERANCES**:

1. Khor B, Gardet A, Xavier RJ. Genetics and pathogenesis of inflammatory bowel disease. Nature. 2011;474(7351):307–17.
2. Ulitsky, A., Ananthakrishnan A.N., Naik A., et al. Vitamin D deficiency in patients with inflammatory bowel disease: Association with disease Activity and quality of life. Journal of parenteral and Enteral nutrition 2011;35:308_316
3. Kong J, Zhang Z et al. Novel role of vitamin D receptor in maintaining integrity of intestinal mucosal barrier. Am J Physiology Gastrointestinal liver physiology 294 (1), G208-G216.
4. Ross AC, Manson JE, Abrams SA *et al*. The 2011 Report on dietary reference intakes for calcium and vitamin D from the Institute of Medicine: what clinicians need to know. *J Clin Endocrinol Metab* 2011; 96: 53–58.
5. Andjelkovic, Z., Vojinovic, J., Pejnovic, N., Popovic, M., Dujic, A., Mitrovic, D. et al. (1999) Disease modifying and immune modulatory effects of high dose 1 alpha (OH) D3 in rheumatoid arthritis patients. Clin Exp Rheumatol 17: 453_456.
6. Nordvik, I., Myhr, K.M., Nyland, H. and Bjerve, K.S. (2000) Effect of dietary advice and n-3 supplementation in newly diagnosed MS patients. *Acta Neurol Scand* 102: 143_149.
7. Goldberg, P., Fleming, M.C. and Picard, E.H. (1986) Multiple sclerosis: Decreased relapse rate through dietary supplementation with calcium, magnesium and vitamin D. Med Hypotheses 21: 193_200.
8. Joseph AK, et al. 25(OH) vitamin D level in Crohn’s disease: association with sun exposure and disease activity 2009.    *Indian J Med Research* 130: 133-137
9. Miheller et al. Comparison of the Effects of 1,25 Dihydroxyvitamin D and 25 Hydroxyvitamin D on Bone Pathology and Disease Activity in Crohn’s Disease Patients. Inflamm Bowel Dis 2009;15:1656–1662.
10. Jorgenson S.P et al. Clinical trial: vitamin D3 treatment in Crohn’s disease – a

Randomized double-blind placebo-controlled study. Aliment Pharmacol Ther 2010; 32: 377–383

1. Ross AC, Manson JE, Abrams SA *et al*. The 2011 Report on dietary reference intakes for calcium and vitamin D from the Institute of Medicine: what clinicians need to know. *J Clin Endocrinol Metab* 2011; **96**: 53–58.
2. [Irvine EJ](https://vpn.ucsf.edu/,DanaInfo=www.ncbi.nlm.nih.gov+pubmed?term=%22Irvine%20EJ%22%5BAuthor%5D), [Zhou Q](https://vpn.ucsf.edu/,DanaInfo=www.ncbi.nlm.nih.gov+pubmed?term=%22Zhou%20Q%22%5BAuthor%5D), [Thompson AK](https://vpn.ucsf.edu/,DanaInfo=www.ncbi.nlm.nih.gov+pubmed?term=%22Thompson%20AK%22%5BAuthor%5D). The Short Inflammatory Bowel Disease Questionnaire: a quality of life instrument for community physicians managing inflammatory bowel disease. CCRPT Investigators. Canadian Crohn's Relapse Prevention Trial. [Am J Gastroenterol.](https://vpn.ucsf.edu/pubmed/,DanaInfo=www.ncbi.nlm.nih.gov+8759664) 1996 Aug;91(8):1571-8. Department of Medicine, McMaster University, Hamilton, Ontario.
3. Vieth, R. [Vitamin D supplementation, 25-hydroxyvitamin D concentrations, and safety](http://www.ncbi.nlm.nih.gov/pubmed/10232622). Am J Clin Nutr. 1999 May; 69 (5): 842-56.
4. Koutkia, P. Chen, T. C. Holick, M. F. [Vitamin D intoxication associated with an over-the-counter supplement](http://www.ncbi.nlm.nih.gov/pubmed/11439958). N Engl J Med. 2001 Jul 5; 345 (1): 66-7.
5. Los Angeles Times [Supplements guru sues over his own product](http://latimesblogs.latimes.com/booster_shots/2010/04/supplements-guru-sues-over-his-own-product.html). 2010/4/29;
6. Jones, G. [Pharmacokinetics of vitamin D toxicity](http://www.ncbi.nlm.nih.gov/pubmed/18689406). Am J Clin Nutr. 2008 Aug; 88 (2): 582S-586S.
7. Heaney, R. P. [Vitamin D: criteria for safety and efficacy](http://www.ncbi.nlm.nih.gov/pubmed/18844846). Nutr Rev. 2008 Oct; 66 (10 Suppl 2): S178-81.
8. Vieth, R. [Vitamin D toxicity, policy, and science](http://www.ncbi.nlm.nih.gov/pubmed/18290725). J Bone Miner Res. 2007 Dec; 22 Suppl 2V64-8.
9. Vieth, R. [Critique of the considerations for establishing the tolerable upper intake level for vitamin D: critical need for revision upwards](http://www.ncbi.nlm.nih.gov/pubmed/16549491). J Nutr. 2006 Apr; 136 (4): 1117-22
10. NIH Office of Dietary Supplements [Dietary Supplement Fact Sheet: Vitamin D](http://ods.od.nih.gov/factsheets/vitamind/). 2009/11/13;
11. Deluca, H. F. Prahl, J. M. Plum, L. A. [1,25-Dihydroxyvitamin D is not responsible for toxicity caused by vitamin D or 25-hydroxyvitamin D](http://www.ncbi.nlm.nih.gov/pubmed/20965147). Arch Biochem Biophys. 2010 Oct 18;
12. Schroeder KW, Tremaine WJ, Ilstrup DM. Coated oral 5-aminosalicylic acid therapy for mildly to moderately active ulcerative colitis: a randomized study. N Engl J Med 1987;317:1625-9.
13. Best WR, Becktel JM, Singleton JW, Kern F Jr. (Mar 1976). "Development of a Crohn's disease activity index. National Cooperative Crohn's Disease Study". *Gastroenterology* **70** (3): 439–444.
